# Porous Ti_3_C_2_T_*x*_ MXene Membranes for Highly Efficient Salinity Gradient Energy Harvesting

**DOI:** 10.1021/acsnano.1c08347

**Published:** 2022-01-09

**Authors:** Seunghyun Hong, Jehad K. El-Demellawi, Yongjiu Lei, Zhixiong Liu, Faisal Al Marzooqi, Hassan A. Arafat, Husam N. Alshareef

**Affiliations:** § Materials Science and Engineering, Physical Science and Engineering Division, King Abdullah University of Science and Technology, Thuwal 23955, Saudi Arabia; ‡ Center for Membranes and Advanced Water Technology, Department of Chemical Engineering, Khalifa University, Abu Dhabi 127788, United Arab Emirates

**Keywords:** titanium carbide, lamellar structured membranes, chemical nanopore etching, nanoconfined fluidic channels, salinity gradient power generation

## Abstract

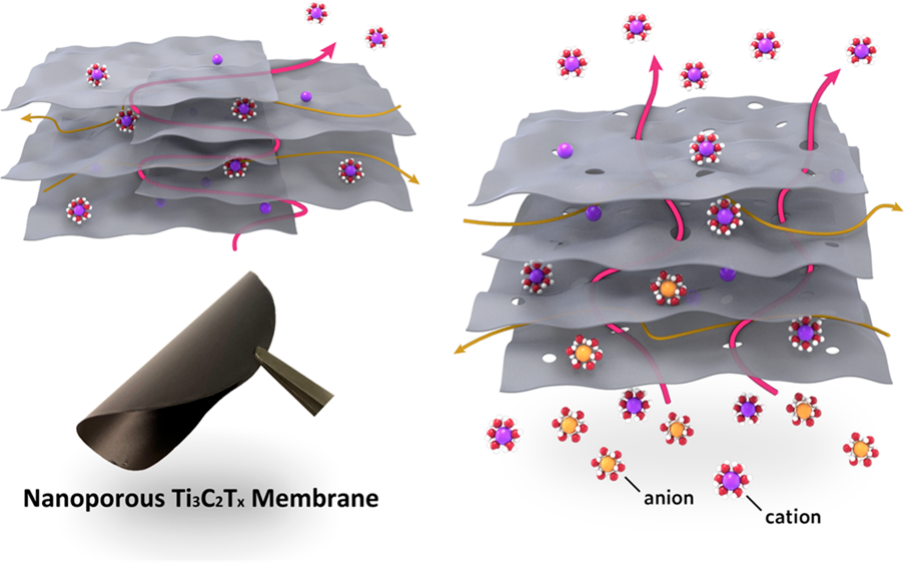

Extracting osmotic energy through nanoporous membranes is an efficient way to harvest renewable and sustainable energy using the salinity gradient between seawater and river water. Despite recent advances of nanopore-based membranes, which have revitalized the prospect of blue energy, their energy conversion is hampered by nanomembrane issues such as high internal resistance or low selectivity. Herein, we report a lamellar-structured membrane made of nanoporous Ti_3_C_2_T_*x*_ MXene sheets, exhibiting simultaneous enhancement in permeability and ion selectivity beyond their inherent trade-off. The perforated nanopores formed by facile H_2_SO_4_ oxidation of the sheets act as a network of cation channels that interconnects interplanar nanocapillaries throughout the lamellar membrane. The constructed internal nanopores lower the energy barrier for cation passage, thereby boosting the preferential ion diffusion across the membrane. A maximum output power density of the nanoporous Ti_3_C_2_T_*x*_ MXene membranes reaches up to 17.5 W·m^–2^ under a 100-fold KCl gradient at neutral pH and room temperature, which is as high as by 38% compared to that of the pristine membrane. The membrane design strategy employing the nanoporous two-dimensional sheets provides a promising approach for ion exchange, osmotic energy extraction, and other nanofluidic applications.

## Introduction

Climate change is becoming a very significant threat, rapidly expanding to all aspects of life.^1^ Fossil fuels are considered as the primary culprit behind this unprecedented climate change. In this perspective, alternative energy sources have been extensively explored to meet the growing global energy demand while minimizing the impact on the environment.^2^ Among the existing renewable energy resources, osmotic energy released from the mixing of aqueous streams with a salinity gradient has attracted considerable attention as a renewable and sustainable source of energy in the past decade.^3−6^ In principle, harnessing osmotic power follows the Gibbs free energy of mixing, where the electric currents could be directly scavenged using reverse electrodialysis (RED). The latter has recently witnessed significant progress due to the advancements in nanostructured membrane fabrication.^7−9^ In a RED operation, ion-exchange membranes bearing preferential counterion diffusion play a key role in energy conversion; however, conventional semipermeable membranes show limited power density due to their high internal resistance.

To date, a wide range of nanomaterials has been exploited for harvesting the ionic gradient energy, including metal–organic frameworks (MOF),^10^ boron nitride nanotubes (BNNT),^11^ and nanoporous molybdenum disulfide (MoS_2_).^12^ The nanoscale pores or channels in these nanostructures could boost both ionic conductance and charge selectivity. For instance, a single-layer MoS_2_ nanopore yielded a power density of up to 1 MW·m^–2^, several orders of magnitude higher than previously reported membranes. This performance is attributed to ultrahigh ionic conductance across the atomically thin layer.^12^ However, despite its outstanding energy conversion outperforming conventional ion-exchange membranes, several technical barriers to its fabrication still hinder its application to a full-scale system.

In this regard, two-dimensional (2D) layered membranes, which can be formed by restacking 2D materials, have been proven a scalable alternative to harvest the osmotic energy. Slit-shaped 2D conduits formed in between neighboring sheets offer subnanometer-scale fluidic channels, facilitating surface-charge-governed ion diffusion. This fascinating feature could be well demonstrated by various planar nanomaterials, *e.g.*, graphene oxide,^13−16^ carbon nitride,^17^ boron nitride,^18,19^ vermiculite,^20^ and most recently, the fast-growing family of MXene.^21−23^ However, despite the increasing interest in scalable lamellar membranes for blue energy harvesting, a rational design strategy is still sought to overcome several coexisting challenges, such as the prolonged ion-diffusion pathways and derived sluggish fluidic transport arising from the restacking and agglomeration of 2D sheets.^23,24^ In particular, with regard to the former hurdles, developing lamellar structures that can explicitly promote faster transversal ion-diffusion (*i.e.*, across the interlayer spacing) is more impactful, given that in-plane diffusion is known to be much slower than out-of-plane diffusion. From this perspective, using lamellar membranes made of nanoporous (hole-etched) 2D sheets stands out as a potential approach. The interplanar channels created by stacks of nonporous (pristine) 2D sheets typically allow for an inertia flow and a vertical flow around the edges or junctions of the sheets.^25,26^ However, the length of the ion diffusion pathway would be much longer than that of ion diffusion directly through the nanoporous sheets. The perforated holes in the basal plane of the nanoporous 2D sheets can effectively create shortened and continuous charge-transport pathways for faster ion transport across the lamellae structures. Thus, benefiting from the advantages of both 2D-layered and nanoporous architectures, typical nanoporous 2D material is an attractive scaffold for constructing ion channels that offer highly selective and rapid transport under salinity gradient. In addition, the nanoporous sheet effectively alleviates the restacking issue when fabricating membranes thick enough to ensure high mechanical stability.^27^

Among the existing 2D materials, MXene (a new class of transition metal carbide, nitrides, or both) provides an appealing framework for lamellar membranes. The layered structure of MXenes, coupled with their surface hydrophilicity, can hold water molecules in between the neighboring sheets, forming fluidic conduits for the conveyance of both ions and molecules. Consequently, MXene membranes can form densely interconnected interplanar nanocapillaries with subnanometer features.^26,28−30^ Recently, Ti_3_C_2_T_*x*_ (*i.e.*, the most studied MXene by far), where T_*x*_ denotes a group of surface terminal species (−Cl, −F, −OH, and =OH), has already demonstrated its potential for osmotic energy harvesting.^21,22,31^ Nevertheless, the attained osmotic power density could be further improved if perforated MXene sheets are employed. In such a case, the etched holes surrounded by surface-terminated functional groups could serve as cation channels without compromising ion selectivity.^32^ Consequently, the nanoconfined internal pores could contribute to the enhancement of the generated power.

Herein, we report scalable lamellar membranes fabricated by restacking nanoporous 2D Ti_3_C_2_T_*x*_ MXene sheets as a nanofluidic platform for high-performance osmotic power generation. The nanosized holes are intentionally introduced into the MXene sheets *via* facile and H_2_SO_4_-based scalable etching method. The nonporous-to-porous transition, using a mild acid oxidizer, *i.e.*, H_2_SO_4_, neither deteriorates the crystallinity nor affects the surface functionality of the unetched parts of the Ti_3_C_2_T_*x*_ sheets. With this approach, the nanoporous MXene lamellar membranes were able to overcome the trade-off between permeability and selectivity, exhibiting much enhanced osmotic power than that obtained by the pristine membranes. Such an augmented osmotic diffusion is supported by the formed lower energy barrier for ion penetration. Moreover, nanoporous Ti_3_C_2_T_*x*_ MXene membranes with higher packing density exhibit prolonged stability over a 100 h operation as well as in a seawater-simulated conditions. The performance of our nanoporous lamellar structures shows a viable path to high-efficiency osmotic energy conversion through MXene-based membranes.

## Results and Discussion

**Figure 1:**
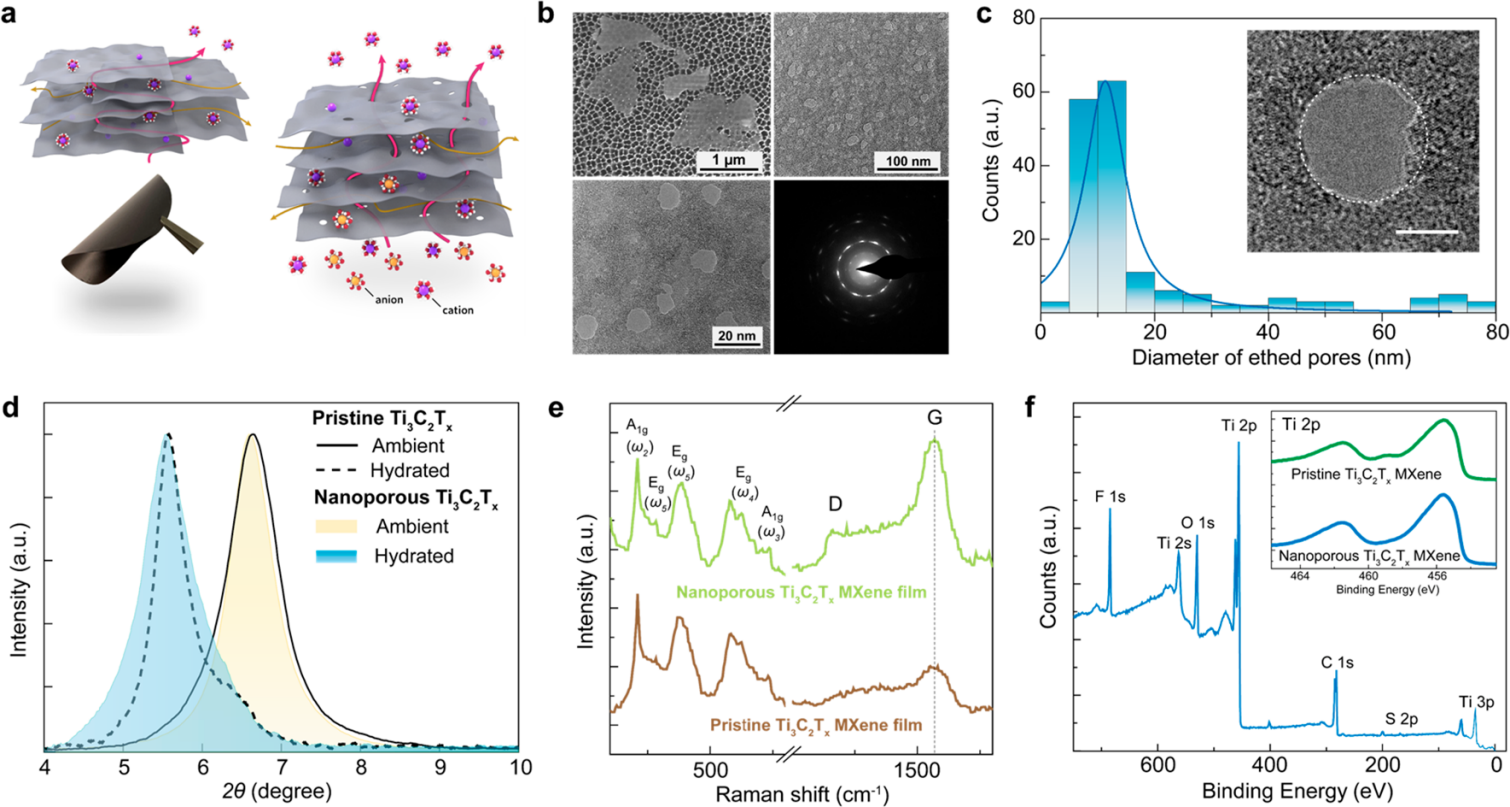
Properties of the nanoporous Ti_3_C_2_T_*x*_ MXene and its derived lamellar membranes (a) Schematic of ion transport through the chemically etched nanopore and two-dimensional slit channels in lamellar MXene membranes. Also shown is a photograph of the reconstructed lamellar nanoporous MXene membrane. (b) SEM, TEM and SAED images of nanoporous Ti_3_C_2_T_*x*_ sheets. (c) Size distribution of etched holes on the Ti_3_C_2_T_*x*_ sheets. Inset: single etched-hole with well-retained crystal structures around the hole (scale bar: 5 nm). (d) X-ray diffraction patterns of lamellar membranes, constructed by pristine and hole-etched sheets, under ambient conditions and in the fully hydrated state. Inset: cross-sectional SEM analysis of nanoporous MXene lamellar membrane (scale bar: 5 μm). (e) Raman spectra of pristine and hole-etched Ti_3_C_2_T_*x*_ sheets. (f) XPS survey spectrum of the hole-etched Ti_3_C_2_T_*x*_ sheets.

Ti_3_C_2_T_*x*_ MXene sheets were synthesized by selectively etching Al atoms from MAX phase Ti_3_AlC_2_ using an HF/HCl etchant followed by a lithium ion-based delamination process, as previously reported.^33−36^ Afterward, hole-etched MXene sheets were obtained by admixing the aqueous suspension of as-synthesized Ti_3_C_2_T_*x*_ sheets with 3 mol·L^–1^ H_2_SO_4_ at an equivolume ratio, which was then left to bake inside a vacuum oven^34^ (see details in the Experimental Methods). Free-standing lamellar membranes made of both pristine and hole-etched Ti_3_C_2_T_*x*_ sheets were, respectively, fabricated using vacuum filtration assembly. A photograph and a schematic depiction of the fabricated nanoporous MXene membranes is displayed in Figure1a. In general, the stacked structure of two-dimensional sheets only allows for limited and mostly lengthy fluidic pathways across the thickness of the membranes. The length of a single capillary extending through the lamellar membranes, involving the fluidic turns and sheet size, could be several thousand times longer than the thickness of membrane itself.^21,37^ As a result, the holes etched throughout the lamellar membranes, serving as a shortcut, can significantly enhance the ionic transport and induce faster charge-selective ion diffusion.

Figure 1b displays the SEM images of individual Ti_3_C_2_T_*x*_ sheets with etched holes and the magnified TEM images of the holes. The etched holes are mostly circular in shape, and the two-dimensional crystalline structure could be confirmed from the observed diffraction pattern as well as the restacked lamellar arrangement even after the acidic etching process. Moreover, from the hole-size distribution analysis in Figure 1c, most of the synthesized holes have diameters in the range of 5–15 nm. As further displayed in Figure S1, the acidic etching of atomically thick holes through the MXene sheets is proven effective, and the estimated density of pores is approximately 10^10^ cm^–2^. It is worth noting that even after the chemical etching process the pristine areas of the sheets surrounding the etched holes have retained their crystallinity as well as their surface functional groups attracting counterions.

Figure 1d demonstrates the swelling behaviors following the aqueous hydration of the pristine Ti_3_C_2_T_*x*_ and hole-etched, nanoporous Ti_3_C_2_T_*x*_ lamellar membrane, respectively. The well-defined layered structure exhibited by the nanoporous Ti_3_C_2_T_*x*_ can be confirmed from the X-ray diffraction pattern as well as the corresponding cross-sectional SEM analysis, shown as an inset. In the hydrated state, the stacked nanoporous Ti_3_C_2_T_*x*_ MXene sheets are separated by an interlayer distance (*d*) 15.93 Å. Considering that a theoretical thickness (*a*) of a monolayer Ti_3_C_2_T_*x*_ sheet is ∼9.8 Å, the empty space, which is available for ions to diffuse, is estimated to be δ = (*d* – *a*) ∼ 6.1 Å. This effective interplanar spacing for ion transport corresponds to the height of a nanocapillary. The pristine Ti_3_C_2_T_*x*_ MXene membrane exhibits nearly comparable volumetric expansion in an aqueous solution to that of the nanoporous membrane. As comparatively shown in Figure S2, the pristine Ti_3_C_2_T_*x*_ sheets demonstrate a 2D-like nature and high hexagonal crystallinity. From the size distribution analysis for individual sheets, the averaged lateral size of pristine Ti_3_C_2_T_*x*_ sheets is around 4.24 μm, and the atomic force microscopy profile of a single sheet shows its height in the range of 1.5 to 2.0 nm. The lateral sizes of the nanoporous Ti_3_C_2_T_*x*_ MXene sheets are comparable to those from pristine sheets.

Further, the synthesis quality and surface stoichiometry of our nanoporous Ti_3_C_2_T_*x*_ sheets were probed using Raman spectroscopy and X-ray photoelectron spectroscopy (XPS) (Figures 1e,f and S3). In the Raman spectrum of the nanoporous MXene, the peaks at 210, 282, 383, 624, and 735 cm^–1^ are, respectively, assigned to the vibrational modes as A_1g_ of Ti_3_C_2_O_2_, E_g_ of Ti_3_C_2_(OH)_2_, E_g_ of Ti_3_C_2_O_2_, E_g_ of Ti_3_C_2_(OH)_2_, and A_1g_ of Ti_3_C_2_O_2_.^34,38−42^ Noteworthy, the noticeable increase in the D and G bands, compared to those from the pristine sheet, is possibly due to residual carbon species on the surface.^42^ Excess carbon can be formed during the etching process, where surfacial Ti atoms can be partially oxidized. Nonetheless, as indicated in the high-resolution XPS spectra of the Ti 2p core level of both pristine and hole-etched Ti_3_C_2_T_*x*_, shown in Figure 1f, the inevitable H_2_SO_4_-induced oxidation effect was marginal given the minimal amount of the formed TiO_2_ relative which was initially present in the pristine MXene (inset of Figure 1f). The XPS survey scan confirms the abundant presence of the functional groups (T_*x*_) at the surface of the hole-etched MXene sheets, along with traces of sulfur compounds. The latter could be either chemisorbed or physisorbed on the surface of nanoporous sheets.

**Figure 2:**
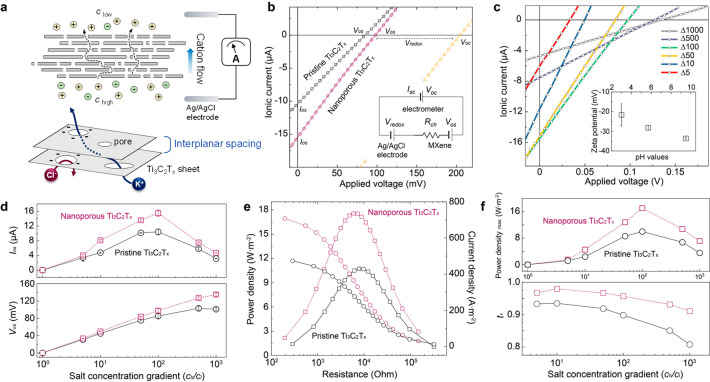
Osmotic power conversion across nanoporous MXene membranes (a) Drift-diffusion experiment across nanoporous Ti_3_C_2_T_*x*_ lamellar membranes under a salt concentration gradient. The cation-selective membrane allows the transport of cations across its etched nanopores and two-dimensional slits, while electrostatically repelling anions, thus building up an electrical gradient across the membrane. (b) Current–voltage characteristics under a 100-fold KCl concentration gradient of (0.5–5) × 10^–3^ mol·L^–1^. Redox reaction arising from unequal chloride concentration at electrodes is subtracted from measured current, and the full red line represents, thus, the pure electroosmotic contribution. Inset: equivalent circuit diagram. (c) *IV* characteristics of nanoporous Ti_3_C_2_T_*x*_ membranes for varying KCl concentration gradient under ambient conditions. Inset demonstrates Zeta potentials of nanoporous Ti_3_C_2_T_*x*_-stacked membranes at varying pH values. (d) Comparative osmotic current and potential of the nanoporous and pristine Ti_3_C_2_T_*x*_ membranes with concentration gradient. Thicknesses of nanoporous and pristine MXene membrane are, respectively, 0.6 and 0.47 μm. (e) Power density and current density as a function of external resistance. (f) Power density and cation selectivity at varying KCl concentration gradients.

To discern the ion-transport properties across the nanoporous Ti_3_C_2_T_*x*_ membranes, we comparatively investigated a current–voltage (*I–V*) transport for the pristine and nanoporous MXene lamellar membranes, respectively, under various KCl concentration gradients. The *I–V* characteristics provided essential information on the impact of created holes on ion diffusive transport across the membranes. The ionic current across the membranes was measured using a pair of Ag/AgCl electrodes as illustrated in Figure 2a. Charge separation across interplanar channels and the etched holes is essential to harvest the electrical energy from the chemical potential gradient. The cation-selective passage toward low from high concentrations, whereas anions are electrostatically retarded, leads to a positive net current across the membranes. Figure 2b shows the representative *I–V* characteristics under a KCl concentration gradient across the pristine and nanoporous Ti_3_C_2_T_*x*_ membrane, respectively. A direction of short circuit current (*I*_*sc*_) in the absence of bias is consistent with a net flow of positive charges, and this charge-selective osmotic flow produces an open-circuit voltage (*V*_oc_) across the membranes. The pure electroosmotic power can then be calculated from osmotic current (*I*_os_) and potential (*V*_os_) by calibration with redox potentials (*V*_redox_) emanating from unequal potential drops at the electrode–solution interfaces in different salt concentration.^43,44^

We investigated the osmotic potentials and currents of the nanoporous Ti_3_C_2_T_*x*_ MXene membranes under a series of KCl concentration gradients. The lower concentrations are in the range of 0.5 × 10^–3^ to 0.1 mol·L^–1^, and the higher concentration is fixed at 0.5 mol·L^–1^ while being in contact with the bare Ti_3_C_2_T_*x*_ membranes. As shown in Figure 2c,d, the osmotic potential increased from 33 to 134 mV at neutral pH by varying the concentration gradients from 10- to 1000-fold. The osmotic current reached up to 15.5 μA under the salt gradient of 100, followed by gradual decline with increasing gradient. Such a drop is likely attributable to strongly developed concentration polarization at the membrane surface of the permeate side. We also explored the current density and power density of the membranes as a function of external resistance under a 100-fold concentration gradient of 0.5 to 5 × 10^–3^ mol·L^–1^ (Figure 2e). Electrical power (*P*) is directly calculated as *P* = *I*^2^ × *R*_m_ where *I* is the measured current and *R*_m_ is the membrane resistance. The extractable power reaches its maximum value (*P*_max_ = 1/4*G*_os_*V*^2^_os_) when the external resistance is equal to the internal resistance of the membrane.^43^ The effective fluidic area is approximately 2.5 × 10^–2^ mm^2^, with a percentage of the overall membrane surface area of less than 0.1% (Figure S4). The current densities decrease with elevating the external load resistance. The nanoporous Ti_3_C_2_T_*x*_ membrane exhibited a maximum output power density of about 17.5 W·m^–2^, which is as high as 38% compared to that of the pristine membrane. The higher current density in the presence of the etched holes should be beneficial in extracting the osmotic power more efficiently.

Figure 2f shows higher maximum power densities generated by the nanoporous membrane than those from the pristine one, under all the applied concentration gradients. In particular, at a 50-fold salinity gradient at which the seawater is mixed with river water, the achieved power density is increased to 12.8 W·m^–2^, well above the benchmark (5 W·m^–2^) for successful osmotic power commercialization. Moreover, the nanoporous membranes exhibited ultrahigh charge selectivity, as indicated by their cation transference number (*t*_*+*_), reaching up to 0.98. The quantity *t*_+_ is calculated as 0.5(1 + *V*_os_/*V*_redox_), and it equals 1 for ideal selectivity and 0.5 for nonselective membrane. The energy conversion efficiency η, calculated as (2*t*_+_ – 1)^2^/2, is as high as 46% under a 10-fold concentration gradient (Figure S5a). The osmotic potential also allows the calculation of the mobility ratio (μ_+_/μ_–_) using the Henderson equation for monovalent species^45,46^
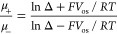
1where *F* is the Faraday constant, *R* is the universal gas constant, *T* = 300 K, and Δ is the ratio of concentration in the feed and permeate solutions. Figure S5b plots the potassium-to-chloride ion mobility ratio calculated using eq 1. The relative mobility ratio for the nanoporous structure were larger than those for the pristine membrane, implying that the pores contribute to rapid and highly charge-selective diffusion.

**Figure 3:**
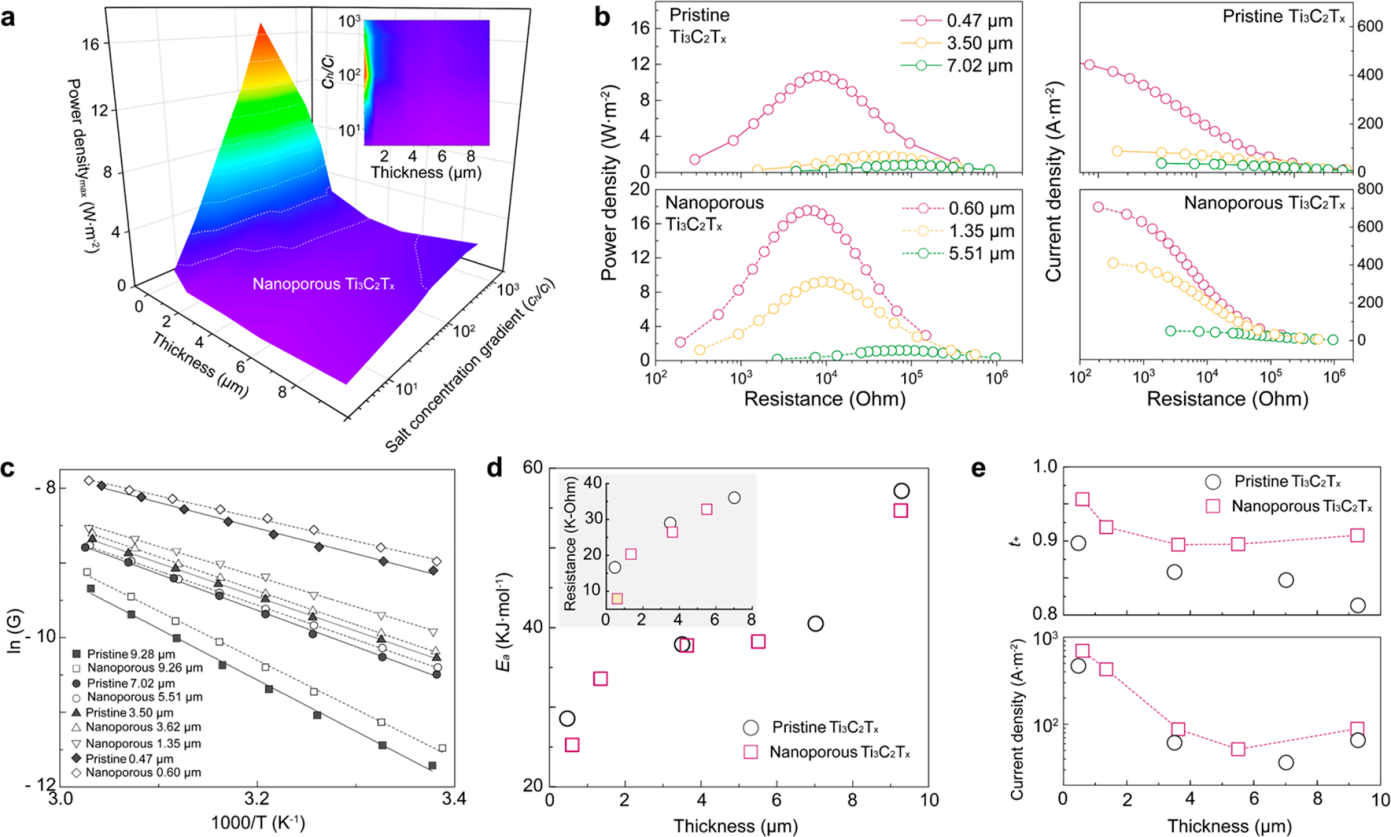
Thickness-dependent osmotic power conversion. (a) 3D Bode maps of maximum osmotic power density-membrane thickness-salt concentration gradient for nanoporous Ti_3_C_2_T_*x*_ MXene membrane; (b) thickness-dependent power density and current density as a function of external resistance; (c) Arrhenius plot of the logarithmic conductance *versus* inverse temperature, obtained from the Ti_3_C_2_T_*x*_ MXene membranes with different thicknesses; (d) energy barrier for K^+^ ion passage at elevated thickness, calculated from the equimolar ionic conductances at different temperatures and pH 5.7 in KCl 10^–2^ mol·L^–1^. The Arrhenius equation was applied, obtainable by plotting the logarithm of conductance against reciprocal of the temperature. Inset: thickness-dependent resistances of both membranes at 296 K, and filled square indicates the nanoporous Ti_3_C_2_T_*x*_ MXene membrane at a thickness of 0.47 μm, deviating from the linear channel resistivity. (e) Thickness-dependent cation transference number and energy conversion efficiency at KCl concentration gradient of 0.5 to 5 × 10^–3^ mol·L^–1^.

Next, to gain insight into the impact of the etched holes on the ionic transportation, we further explored the osmotic transport of the membranes with varying thicknesses and under different salt concentration gradients. From the power density plot illustrated in Figures 3a and S6, both nanoporous and pristine membranes exhibited incremental power densities with decreasing thickness at the elevated concentration gradient. In particular, the output power revealed a relatively strong inverse correlation with the thickness, implying that control over the lamellae channel geometries such as perforated holes can play a critical role in scavenging the osmotic energy. As previously reported from nanopores on atomically thick 2D graphene, boron nitride, or MoS_2_, those ultrathin membranes showed an ultrahigh powder density that is attributable to the extraordinary combination of ionic selectivity and permeability through confined holes. Interestingly, pore etching might help build nanocapillaries with the characteristic length scale (400–1000 nm) of the ideal nanofluidic channel, which was previously reported to optimize osmotic power density while balancing conversion efficiency.^47^ Further reducing the membrane thickness or shrinking the nanosheet dimensions in the presence of perforated holes may provide great power performance. The osmotic power density and current density as a function of external resistance further elucidate that the etched holes on the sheets offer additional shortcut pathways to interconnected 2D channels for ion transportation, leading to lower fluidic resistance across lamellar membranes (Figure 3b).

More specifically, the ionic resistance of a single conduit can be defined by serially combining the respective fluidic resistance for the 2D slit channels, etched pores, and derived nanopore access resistance.^25,48−50^ Corresponding ionic conductance of single channel can be simply expressed as
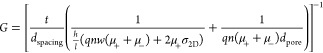
2where *G* is the ionic conductance of single channel across membrane; *t* is the thickness of lamellar membrane; *d*_spacing_ is the interlayer spacing between neighboring sheets; *q* is the elementary charge; *n* is the density of cation or anion; *w*, *l*, and *h*, respectively, stand for the width, length, and effective height of the 2D slit channel; *d*_pore_ indicates the diameter of the perforated pore; μ_+_ and μ_–_ are the mobility of K^+^ and the anion of Cl^–^, respectively; and σ_s_ is the surface charge density of channel. See details in the Supporting Information. As elaborated in Figure S7b, the shortened pathway arising from the created pores obviously contributes to the enhancement in the fluidic conductance compared to that of the nonporous sheet. The only geometric component that impacts ionic conductivity *via* pores is the etched pore diameter. Besides reduced 2D slit channels, which could be linked to the planar size of 2D sheets or the density of perforated pores, can increase ionic conductances significantly. As analytically predicted, geometric adjustments to inner fluidic pores and their associated diameters may be able to further increase osmotic power conversion. The etching duration, reaction temperature, and etchant H_2_SO_4_ concentration can all be adjusted to fine-tune the pores during the wet-etching process.^34,51^ In principle, overall conductance across the lamellar membrane can be derived from equivalent conductance for a parallel combination of the individual channels.

We also investigated the thickness-dependent conductances at elevated temperatures and evaluated the energy barriers for K^+^ transport through the lamellar membranes. As depicted in Figure 3c, the Ti_3_C_2_T_*x*_ membranes displayed ionic conductances linearly dependent on the temperature in the range of 295–330 K and furthermore followed the Arrhenius behavior. The thermal dependence of the membranes is possibly associated with ion mobility enhancement in response to a reduced fluidic viscosity.^52,53^ The energy barrier for K^+^ permeation across the stacked nanoporous Ti_3_C_2_T_*x*_ sheets is revealed to be 25.2 kJ·mol^–1^ at a thickness of 0.6 μm, lower than 28.6 kJ·mol^–1^ for the pristine membrane of 0.47 μm thickness (Figure 3d). This implies that the lower energy barrier from the nanoporous Ti_3_C_2_T_*x*_ membrane, especially below 1 μm thickness, can translate into enhanced osmotic diffusion. The perforated holes through the Ti_3_C_2_T_*x*_ sheets have yielded higher conductances than the pristine membranes at all thicknesses (inset of Figure 3d). Their resistances are definitely decreasing with the thickness. The submicro thin nanoporous membrane shows around 2.7 times higher conductivity of a comparably thin pristine membrane. The increased conductivity of such thin membrane can be explained by the combination of a mainly shorter channels and augmented ion transport routes provided by the etched pores.

More importantly, the perforated pores on the sheets elevate the charge selectivity of the membranes over whole investigated thickness range of ∼0.5 μm to ∼9.3 μm. This implies that the pores inside the lamellar channels can allow higher selective transport without compromising permeability (Figure 3e). We note that excessively high pore density can induce strong ion concentration polarization as well as overlap of charge concentration clouds, which depletes the local concentration gradients across the nanopores and in turn impairs the charge selectivity.^32,54^ Furthermore, the larger the pore size is, the lower the selectivity of a counterion. However, contrasting to the nanopores in contact with electrolytic bulk environment, the ones surrounded by the confined Debye screening layers in between adjacent sheets can work as the interconnected counterion channels through the lamellar Ti_3_C_2_T_*x*_ membrane. Hence, they can boost preferential diffusion of cations. Furthermore, recent studies on the ion selectivity of transmembrane nanopores suggest that the charge selectivity across those pores is mainly governed by the charge separation within the Debye layer formed on the outer charged surfaces rather than the pore walls.^32,55^ The internal pores across the membranes could account for incremental change of the charge-selective transportation, rendering the nanoporous Ti_3_C_2_T_*x*_ superior for osmotic power conversion.

**Figure 4:**
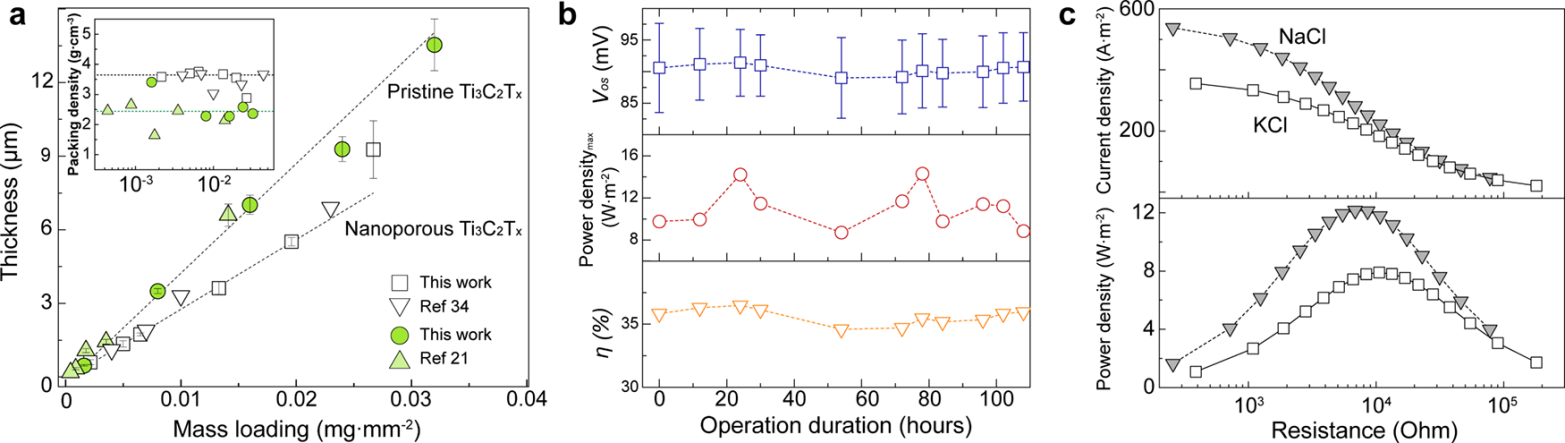
Feasibility of nanoporous MXene-based membranes as osmotic power generator (a) Relationship between mass loading and membrane thickness, yielded from pristine and nanoporous Ti_3_C_2_T_*x*_ membranes. Inset: packing densities of both membranes, derived from the areal mass loading over thickness. (b) Long-term osmotic power conversion in an aqueous KCl electrolyte, measured at pH 5.7 and room temperature over 100 h. (c) Osmotic power generation as a function of external resistance under NaCl concentration gradient. The feasibility evaluation was implemented with nanoporous Ti_3_C_2_T_*x*_ lamellar membrane with 1.35 μm thickness under a KCl and NaCl concentration gradients of 0.5 to 5 mol·L^–1^.

The shortened ion pathway, facilitated by the purposely created pores on the 2D sheets, may offer a structural advantage for fabricating the ion-exchangeable lamellar membranes. In particular, the internal open structure with interconnected transport pathways can significantly alleviate the restacking issues of 2D nanomaterials. Figure 4a shows the relationship between the thickness and the mass loading for the pristine and the nanoporous Ti_3_C_2_T_*x*_ membranes, respectively. The mass loading of the membranes was determined by the total amount of dried Ti_3_C_2_T_*x*_ divided by the area of membrane. Note that the membrane thickness increases linearly with the mass loading.

Interestingly, the nanoporous Ti_3_C_2_T_*x*_ membranes have a packing density of ∼3.5 g·cm^–3^, making it 35% denser than that of pristine membranes. The observed packing densities from both types of membranes are consistent with densities previously reported by others.^21,34^ The dense arrangement morphology in the nanoporous Ti_3_C_2_T_*x*_ MXene filtration film indicates better film formation, which can correlate to the perforated pores *via* a vacuum-assisted trap-and-escape mechanism.^27^ The filtration-induced stacking of pristine Ti_3_C_2_T_*x*_ sheets inevitably forms pockets with water trapped inside. Thus, the slow evaporation through the limited spacing in a subsequent drying processes can result in many voids inside, leading to a relatively lower packing density. In contrast, the etched holes on the individual sheet offer pathway for trapped residual water to escape during the drying process, enabling the formation of tightly packed morphology while avoiding the formation of undesired voids. It is worth mentioning that the nanoporous MXene has retained its highly ordered structure, as evidenced by the relatively sharp and narrow characteristic (002) diffraction peak as shown in Figure S8. Furthermore, the thicker nanoporous Ti_3_C_2_T_*x*_ membranes with 9.26 μm thickness, presumably exerting higher mechanical strength, still exhibit ∼250% higher power densities of about 2.5 W·m^–2^ than those of the thick, pristine Ti_3_C_2_T_*x*_ membranes (Figure S9).

Finally, to assert the advantage of nanoporous Ti_3_C_2_T_*x*_ MXene membranes in practical osmotic power conversion applications, we explored the osmotic transport operation of these membranes over 100 h and evaluated their power conversion performances. As shown in Figure 4b, the nanoporous Ti_3_C_2_T_*x*_ membrane retains a stable osmotic potential of around 90 mV coupled with a derived power conversion efficiency of ∼36% under a 100-fold KCl gradient, with a resulting power density of averaging 9.5 W·m^–2^. The energy conversion efficiency was kept within 3% of the average output power over the course of 108 h. Such stable performance suggests that the nanoporous Ti_3_C_2_T_*x*_ membrane sustained its chemical stability and mechanical integrity even after long-term exposure under an osmotic environment. This physicochemical stability was also probed using X-ray diffraction and Raman spectroscopic studies, as demonstrated in Figure S10. Moreover, this outstanding osmotic power conversion is comparatively investigated using Na^+^, the most abundant ionic species in seawater (Figure 4c). The estimated maximum osmotic power from the seawater-simulated condition (NaCl) reached around 12 W·m^–2^, 54% higher than the corresponding power using KCl concentration gradient. The fluidic improvement might be due to a greater expansion of the interplanar gap, which is associated with a stronger adsorption of sodium ions on Ti_3_C_2_T_*x*_ surfaces.^26^ After extensive exposure to electrolytes, the Ti_3_C_2_T_*x*_ MXene membrane displays a larger quantity of interplanar intercalation for sodium ion than for potassium ion, as previously investigated by X-ray based spectroscopy.^56^ Collectively, these osmotic conversion performances provide a strong prospect of using nanoporous Ti_3_C_2_T_*x*_ MXene membranes for future industrial applications.

## Conclusion

We have developed nanoporous lamellar Ti_3_C_2_T_*x*_ MXene membranes and demonstrated their use in high-performance osmotic power generation. The nanoscale holes with diameters in the range of 5–15 nm could be perforated into 2D Ti_3_C_2_T_*x*_ MXene sheets *via* partial etching by mild acid oxidizer H_2_SO_4_. The etched pores, functioning as interconnected cation channels, have led to osmotic power as high as 17.5 W·m^–2^ under a 100-fold KCl gradient at neutral pH and room temperature, outperforming the pristine Ti_3_C_2_T_*x*_ membrane, as well as other commercially available ion-exchange membranes. The enhancement is strongly associated with concurrently enhanced permeability and selectivity in the presence of an open membrane structure. Furthermore, the nanoporous Ti_3_C_2_T_*x*_ MXene membrane has exhibited excellent long-term structural stability and derived stable energy harvesting performance in aqueous electrolytes. Our findings not only offer a feasible approach to regulate ion transport through the MXene-based membranes but also significantly advance their viability for nanofluidic osmotic power generation.

## Experiemenal Methods

### Synthesis of Nanoporous Ti_3_C_2_T_*x*_ MXene Materials

Ti_3_C_2_T_*x*_ MXene was synthesized by selectively etching Al atoms from a layered ternary MAX-phase Ti_3_AlC_2_ powder with 98 wt % and 400 mesh size (commercially procured from Laizhou Kai Kai Ceramic Materials Co., Ltd.). The etchant was prepared by mixing 3 mL of hydrochloric acid (HCl, Fisher Scientific, technical grade, 35–38%), 3 mL hydrofluoric acid (Sigma-Aldrich, 49%), and 6 mL of cold deionized (DI) water followed by stirring for 5 min. Then 1.2 g of raw Ti_3_AlC_2_ powder was slowly added to the as-prepared etching solution at 40 °C followed by stirring at 550 rpm for 17 h. The acidic suspension (multilayered MXene) was washed several times using centrifugation at 2600 rcf for 5 min per cycle until pH ≥ 6 was reached. Then the sediment was intercalated by 35 mL of 0.8 mol·L^–1^ LiCl at room temperature by stirring for 4 h. The delaminated sediment was washed using DI water *via* centrifugation at 2600 rcf until pH ≥ 6. Further, the sediments containing delaminated single- and few-layer Ti_3_C_2_T_*x*_ MXene sheets were stored in a freezer for 2 h and then redispersed in a small amount of DI water. Highly concentrated supernatant was collected *via* centrifugation at 1000 rcf. Nanoporous MXene sheets were prepared by mixing the suspension of their as-synthesized counterparts with 3 mol·L^–1^ of H_2_SO_4_ at an equivolume ratio, followed by a baking process under a vacuum at 40 °C for 48 h to evaporate the water. The as-obtained Ti_3_C_2_T_*x*_-H_2_SO_4_ slurry was collected and washed using DI water *via* centrifugation at 2600 rcf until pH ≥ 6 was reached. The lamellar Ti_3_C_2_T_*x*_ MXene membranes were fabricated by filtering specific volume of MXene suspension through a polyvinylidene fluoride (PVDF) membrane (0.22 μm pore size and a diameter of 43 mm). The applied concentrations of pristine and nanoporous MXene suspension in water are 1.06 and 0.88 mg·mL^–1^, respectively. All of the prepared lamellar membranes were ambient-dried overnight and could be easily detached from the support.

### Ion-Transport Measurements across Membranes

Ion-transport measurement was carried out using a custom-made electrochemical cell with two reservoirs containing 10 mL each. The free-standing lamellar membranes on a support PVDF membrane (0.2 μm pore size) were placed between the reservoirs. The aperture area for ion penetration is 19.63 mm^2^. To take the current–voltage characteristics across the transmembrane, a pair of Ag/AgCl electrodes connected to a Keithley 2400 sourcemeter was employed to apply voltage and measure the current across membranes. The distance between the electrode and the membrane was constant at 10 mm. The osmotic power was characterized using aqueous KCl solutions of different concentrations ranging from 5 × 10^–3^ to 0.5 mol·L^–1^. The temperature control was implemented by circulating temperature-controlled water through a heating jacket surrounding each reservoir. The temperature of the solutions in each reservoir was monitored by using a Quad MF isoPod system (EPU452, eDAQ) with 1000 Ohm Platinum RTD temperature probes.

### Characterization of Nanoporous Ti_3_C_2_T_*x*_ MXene and Derived Membranes

The microscopic structure of the lamellar membranes was characterized using a field emission scanning electron microscope (Merlin, Carl Zeiss). High-resolution transmission electron microscopic images and a selective area electron diffraction pattern of Ti_3_C_2_T_*x*_ nanosheets were obtained using Titan Cs Image. Atomic force microscopy (Dimension Icon, Bruker) was used to characterize the surface morphology and sheet dimensions of the MXene sheets. The Raman spectra were measured using a Witec alpha 300 confocal Raman microscope equipped with a confocal spectrometer using a 532 nm excitation laser. A typical laser spot is 1–2 μm. The X-ray diffraction (XRD) analysis was carried out using a Bruker D8 Advance with Cu Kα radiation (*l* = 0.15406 nm); the step size was 0.02° with a scan rate of 0.5 step/s. The atomic composition was examined by X-ray photoelectron spectroscopy (XPS) (Axis Ultra DLD, Kratos Analytical). The XPS measurement was performed with a monochromatic Al Kα X-ray source (*hν* = 1486.6 eV) operated at a power of 150 W and under an ultrahigh vacuum in the range of ∼10^–9^ mbar. The zeta potential of MXene dispersions was measured with a Malvern Zetasizer Nano ZS.

## Supporting Information Available

Morphological analysis of etched holes through Ti_3_C_2_T_*x*_ MXene sheets; characterizations of as-synthesized pristine Ti_3_C_2_T_*x*_ MXene sheets; XPS analysis of hole-etched Ti_3_C_2_T_*x*_ MXene sheets; cffective fluidic channels across lamellar MXene membranes; power conversion efficiency and ion-to-ion mobility ratio across membranes; thickness-dependent osmotic power generation at varying concentration gradients; analytical approximation of fluidic resistance across nanoporous channels; X-ray diffraction analysis of membranes with different thicknesses; osmotic power conversion across thick membranes; chemical stability of nanoporous Ti_3_C_2_T_*x*_ MXene membranes under aqueous operation (PDF)
