# Supplementary material for: Porous Ti3C2Tx MXene Membranes for Highly Efficient Salinity Gradient Energy Harvesting
Source: ACS Nano. 2022 Jan 9;16(1):792–800. doi: 10.1021/acsnano.1c08347 (PMC8793134; doi:10.1021/acsnano.1c08347)
Supplement: Supplementary file 1 — nn1c08347_si_001.pdf [file nn1c08347_si_001.pdf]

Supporting Information

Porous $Ti_3C_2T_x$ MXene Membranes for Highly Efficient Salinity Gradient Energy Harvesting

Seunghyun Hong, Jehad K. El-Demellawi, Yongjiu Lei, Zhixiong Liu, Faisal Al Marzooqi, Hassan A. Arafat and Husam N. Alshareef *

* Correspondence and requests for materials should be addressed to Prof. Husam N. Alshareef (Email: husam.alshareef@kaust.edu.sa)

Table of Contents

1. Morphological analysis of etched holes through $Ti_3C_2T_x$ MXene sheets
2. Characterizations of as-synthesized pristine $Ti_3C_2T_x$ MXene sheets
3. XPS analysis of hole-etched $Ti_3C_2T_x$ MXene sheets
4. Effective fluidic channels across lamellar MXene membranes
5. Power conversion efficiency and ion-to-ion mobility ratio across membranes
6. Thickness-dependent osmotic power generation at varying concentration gradients
7. Analytical approximation of fluidic resistance across nanoporous channels
8. X-ray diffraction analysis of membranes with different thicknesses
9. Osmotic power conversion across thick membranes
10. Chemical stability of nanoporous $Ti_3C_2T_x$ MXene membranes under aqueous operation

1. Morphological analysis of etched holes through $Ti_3C_2T_x$ MXene sheets

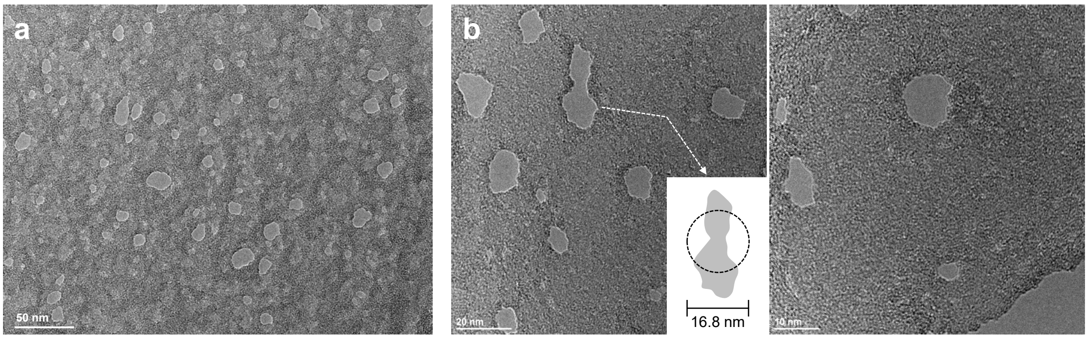

Figure S1 (a) TEM morphologies of chemically etched nanopores in the $Ti_3C_2T_x$ MXene sheets. Note the well-retained crystal structures around the hole. **(b)** Magnified TEM images of the etched holes. The grey outline in the inset is traced from the TEM image, and the diameter of the black dashed circle is estimated from the area of the irregular-shaped hole.

2. Characterizations of as-synthesized pristine $Ti_3C_2T_x$ MXene sheets

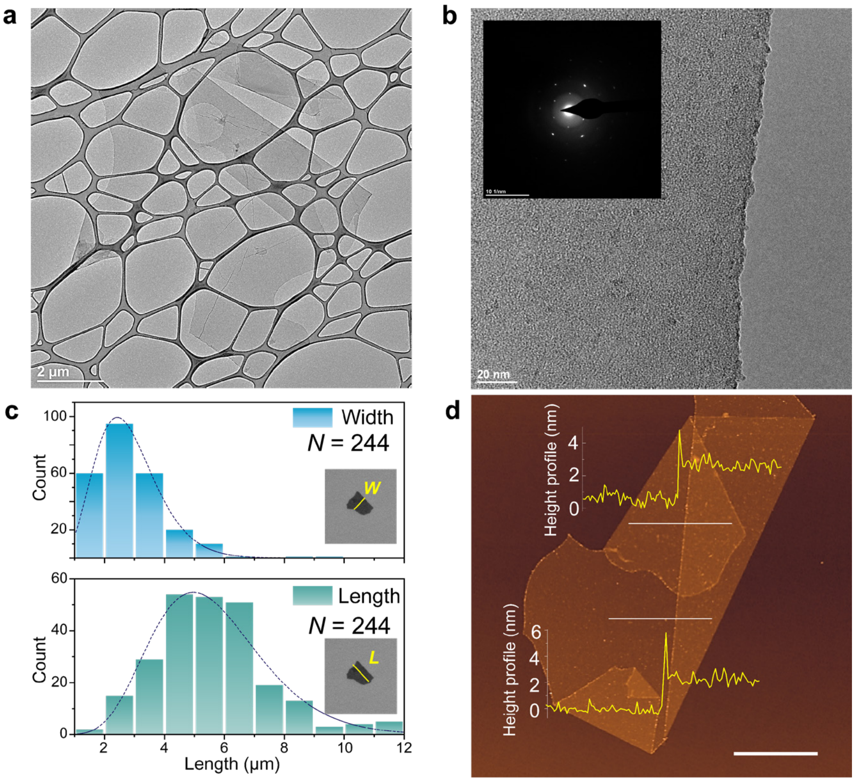

Figure S2 (a-b) TEM images of individual $Ti_3C_2T_x$ MXene sheets on lacey carbon and along with the corresponding selected area electron diffraction (SAED) pattern. **(c)** Size distribution of $Ti_3C_2T_x$ MXene sheets, investigated using SEM over 244 sheets. The width and length of all the sheets deposited on Si/SiO_2 substrates and porous AAO membranes, respectively, are analyzed for the size analysis. **(d)** Topological mapping of a single layer $Ti_3C_2T_x$ nanosheet deposited on a Si/SiO_2 substrate, investigated by AFM (Scale bar: 2 μm). A sheet-to-sheet procedure from the overlapped region was used to determine the thickness of the single nanosheets to exclude instrumental artifacts or the influence of possible contaminants on substrates.

3. XPS analysis of hole-etched $Ti_3C_2T_x$ MXene sheets

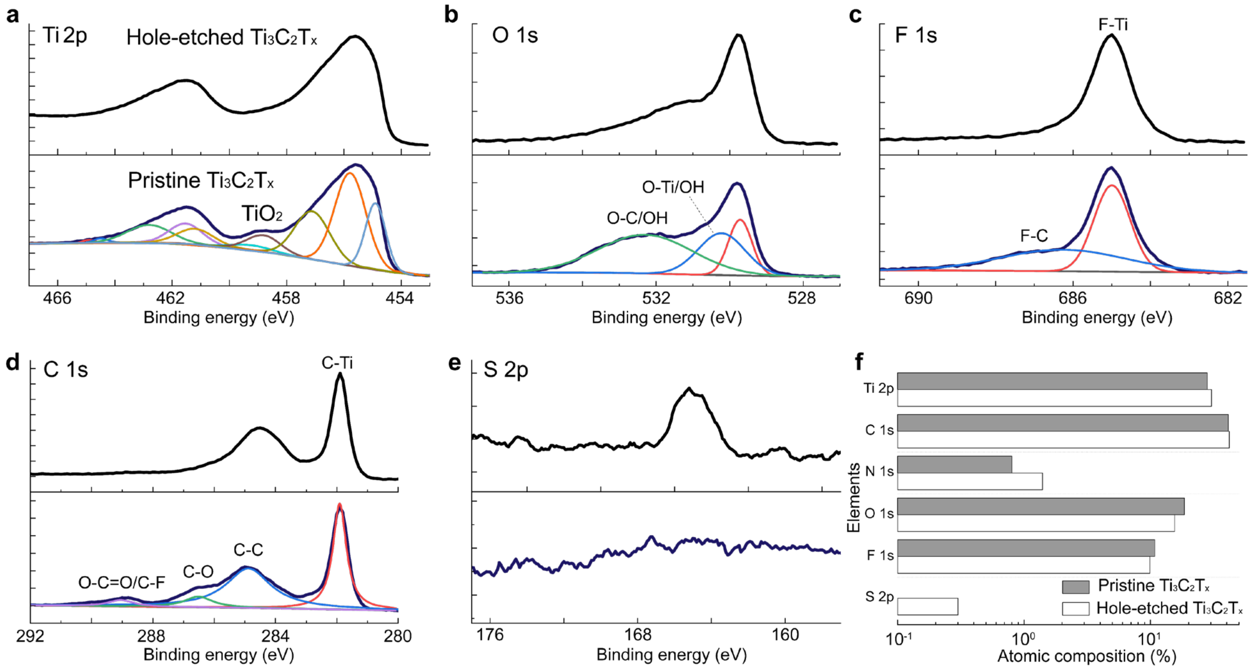

Figure S3 Comparative XPS analysis of membranes made of as-synthesized and hole-etched $Ti_3C_2T_x$ sheets, respectively. Deconvoluted spectra for regions of **(a)** Ti 2p, **(b)** O 1s, **(c)** F 1s, **(d)** C 1s, and **(e)** S 2p, obtained from the stacked pristine and hole-etched $Ti_3C_2T_x$ membranes, respectively. The survey spectra show four possible moieties of surface termination, confirming the presence of oxygen-containing termination. In addition, the residue of sulfur compounds was detected following etching process. **(f)** Atomic composition of the pristine and hole-etched $Ti_3C_2T_x$ sheets, respectively, investigated by high-resolution XPS.

4. Effective fluidic channels across lamellar MXene membranes

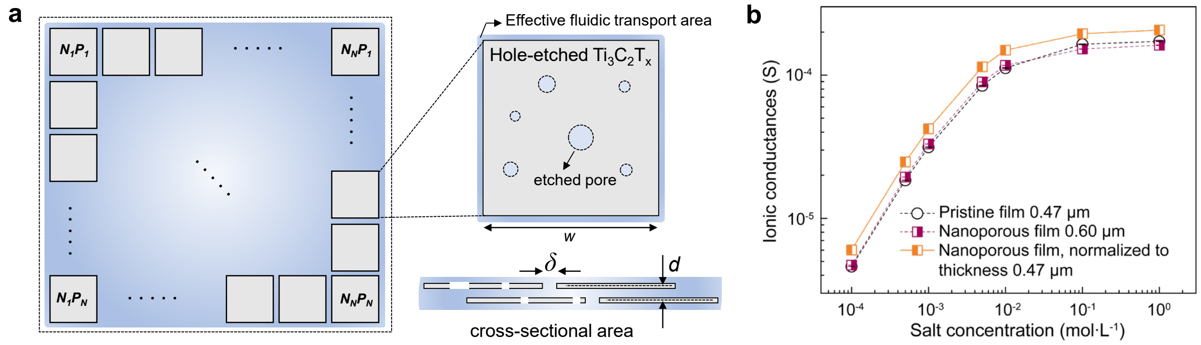

Figure S4 (a) Effective ion transport area across the nanoporous $Ti_3C_2T_x$ lamellar membrane **(b)** Salt concentration-dependent conductances across the pristine and nanoporous $Ti_3C_2T_x$-stacked membranes, respectively.

We assumed that the single $Ti_3C_2T_x$ sheet possesses the same width and length, which are approximated from experimentally averaged lateral sizes of the MXene sheets. The total number of parallel 2D channels per unit area can be estimated as by ~ $2N(N+1)$, and the resulting number of channels is ~ 10^7 across the employed lamellar membranes with a full area of 78.54 mm^2. The effective fluidic area is approximately 2.5×10^{-2} mm^2, and the corresponding fraction of the nanochannels on the total membrane area is estimated to be less than 0.1 % (**Figure** S4a). The perforated holes are responsible for geometric alterations in penetrating fluidic channels, changing the intrinsic property of membranes. As displayed in **Figure** S4b, the improvement from the nanoporous membrane, normalized to the thickness, is

attributable to its increased ion conductivity of the membrane in the presence of pores. Notably, at high salt concentration of 1 mol·L^{-1}, governed by the bulk transport, *i.e.*, directly determined by the given channel geometries rather than surface charges, the change of channel geometries is evidently verified by the observed higher conductance of the nanoporous membrane. The higher conductivity can be explained mostly by a combination of shorter channels and augmented ion transport routes provided by the etched pores. The fraction of the effective transport area is likely to be comparable to that of the pristine membrane. The resultant higher porosity also leads to increased cation selectivity of membranes. The perforated holes might act as cation channels, linking 2D interplanar channels produced between adjacent sheets in lamellar membranes. The internal pores lower the energy barrier for cation passage, thereby boosting the preferential ion diffusion across the membrane.

5. Power conversion efficiency and ion-to-ion mobility ratio across membranes

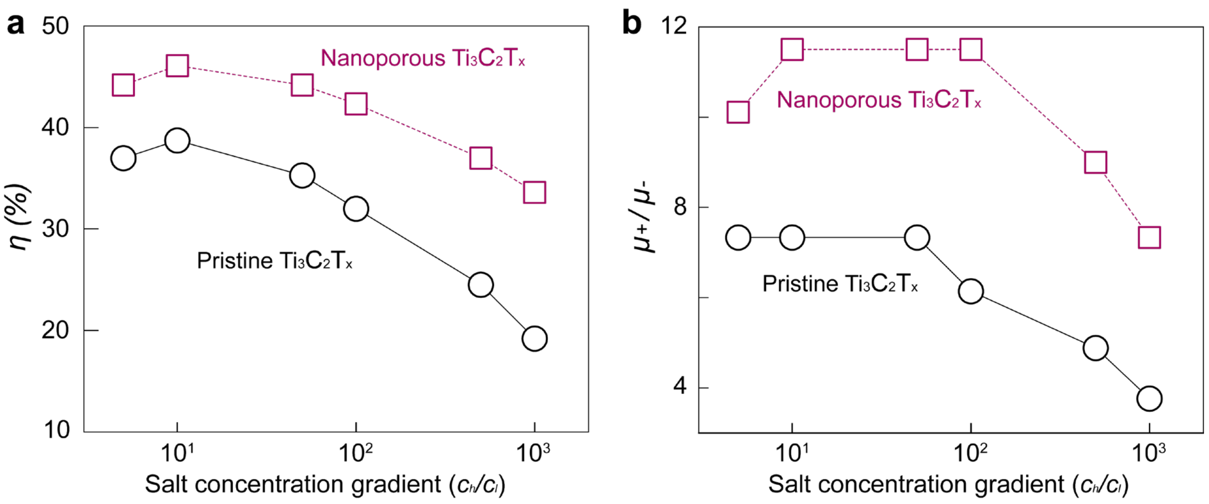

Figure S5 Comparative study on the osmotic power conversion as a function of a salt concentration gradient **(a)** energy conversion efficiency and **(b)** relative ionic mobility ratio across the nanoporous and the pristine $Ti_3C_2T_x$ MXene membranes, respectively.

6. Thickness-dependent osmotic power generation at varying concentration gradients

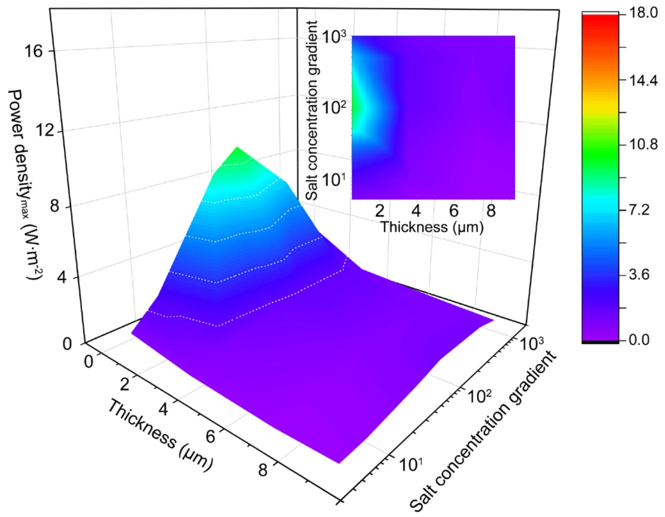

Figure S6 3D Bode maps of maximum osmotic power density-membrane thickness-salt concentration gradient for the pristine $Ti_3C_2T_x$ MXene membrane.

7. Analytical approximation of fluidic resistance across nanoporous channels

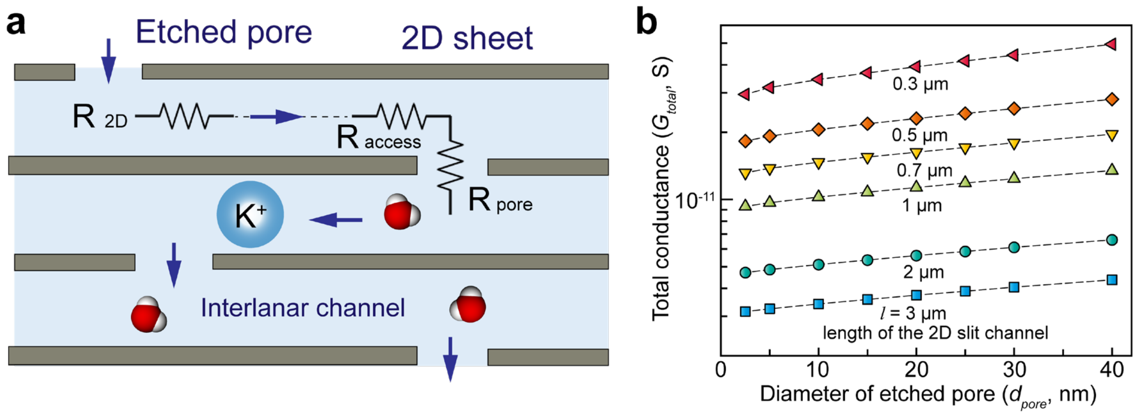

Figure S7 (a) analytical model for ionic penetration through the interconnected channels of 2D interplanar slit and perforated pore. **(b)** Ionic conductances obtained at variable length of nanocapillary and diameter of pore, respectively. Here, the surface charge density is presumed to be 0.1 $C\cdot m^{-1}$ in accordance with previous study.[1] The ionic concentration at 10^{-2} $mol\cdot L^{-1}$ is employed for approximate evaluation.

To understand the impact of geometric factors on total conductances, we define a single conduit by serially combining a two-dimensional interplanar slit and etched pores with varying diameters. The ionic resistance (R_{2D}) through the interplanar spacing in between neighboring sheets can be approximately described by [2]

$$R_{2D} = \left[\frac{h}{l}\left(qnw(\mu_+ + \mu_-) + 2\mu_+\sigma_{2D}\right)\right]^{-1} \quad \text{(S1)}$$

where q is the elementary charge; n is the density of cation or anion; μ_+ and μ_- are mobility of cation (7.62×10^{-8} $m^2\cdot V^{-1}s^{-1}$ for K^+) and anion (7.91×10^{-8} $m^2\cdot V^{-1}s^{-1}$ for Cl^-), respectively; σ_{2D} is the surface charge density of 2D sheets; w, h, and l are, respectively, width, effective height (0.61 nm) and length of the 2D slit channel. The width of 2D slit is assumed to be identical of diameter of etched pores. Note that the left term indicates a bulk conductance, linearly dependent on the salinity, and the right term is a contribution from the electro-osmotic flow of excess counterions, typically described as surface-charge-governed transport.

The pore resistance (R_{pore}) through the etched pore on the basal plane can also be demonstrated with analogous contribution from bulk behavior and confined electrical double layers as [3]

$$R_{pore} = \left[\frac{\pi}{4}\left(\frac{d_{pore}}{L_{pore}}\right)^2\left(qn(\mu_+ + \mu_-) + \mu_+\frac{4\sigma_{pore}}{d_{pore}}\right)\right]^{-1} \quad \text{(S2)}$$

where d_{pore} and L_{pore} are the diameter and length of the etched pore, respectively, and σ_{pore} is the surface charge density of pore. Recent studies on the ion selectivity of transmembrane nanopores suggest that the charge selectivity across the pores is mainly governed by the charge separation within the Debye layer formed on the outer charged surfaces rather than the inner walls of the pores. Even after the chemical etching process, the sheets surrounding the holes remain their crystallinity and presumably surface functional groups attracting counterions. Furthermore, contrary to the nanopores open to electrolytic bulk environment, the nanopores surrounded by the confined Debye screening layers in between adjacent sheets can work as the interconnected counterion channels in the lamellar $Ti_3C_2T_x$ membrane, and possibly boosting preferential diffusion of cations. Consequentially, the surface charge density inside the pores is assumed to be same as that (0.1 $C\cdot m^{-1}$) of the 2D $Ti_3C_2T_x$ sheets in accordance with previous study.

Aside from the resistances through the open structures, the access resistance (R_{acess}) through the atomically thin nanopore is another factor that strongly influences the ionic resistance, as shown by [4]

$$R_{access} = \left[qn(\mu_+ + \mu_-)d_{pore}\right]^{-1} \quad (S3)$$

Ionic conductance of single conduit can be approximately estimated by serially connecting respective resistance as

$$G_{total} = \left[R_{2D\ slit} + R_{pore} + R_{access}\right]^{-1} \quad (S4)$$

We have employed equation (S4) to calculate the ionic conductances through the interconnected channels of 2D interplanar slit and perforated pore. In general, we found that the access resistance governs total conductance through the etched pore, which suggests that pore diameter is the only geometric factor that influences the conductance through the pore. Taking this into account, the total ionic conductance of single fluidic channel across membrane is shown by equation S5:

$$G = \left[\frac{t}{d_{spacing}}\left(\frac{1}{\frac{h}{l}(qnw(\mu_+ + \mu_-) + 2\mu_+\sigma_{2D})} + \frac{1}{qn(\mu_+ + \mu_-)d_{pore}}\right)\right]^{-1} \quad (S5)$$

where t is the thickness of lamellar membrane; $d_{spacing}$ is the interlayer spacing between neighboring sheets. Overall conductance across the lamellar membrane can be derived from equivalent conductance for a parallel combination of the individual channels.

8. X-ray diffraction analysis of membranes with different thicknesses

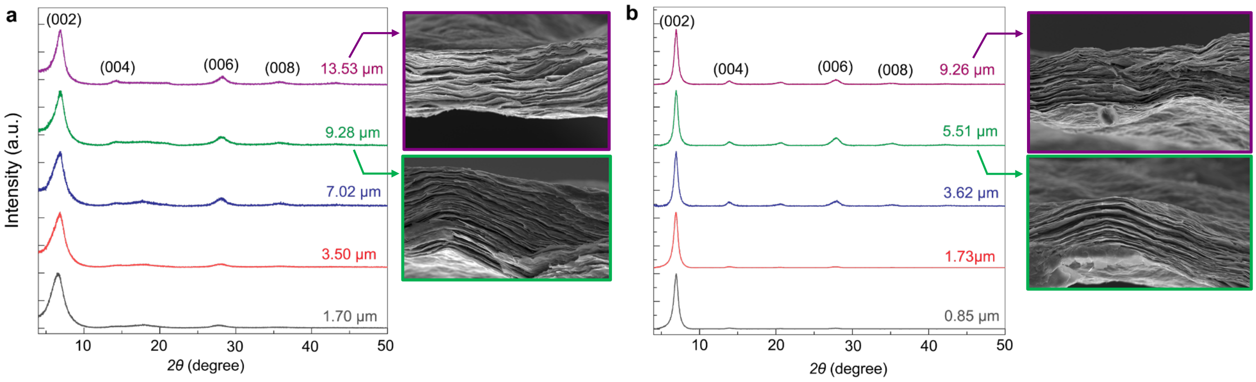

Figure S8 XRD patterns and corresponding cross-sectional SEM images of the **(a)** pristine and **(b)** nanoporous $Ti_3C_2T_x$ MXene membranes, respectively, with different thicknesses.

9. Osmotic power conversion across thick membranes

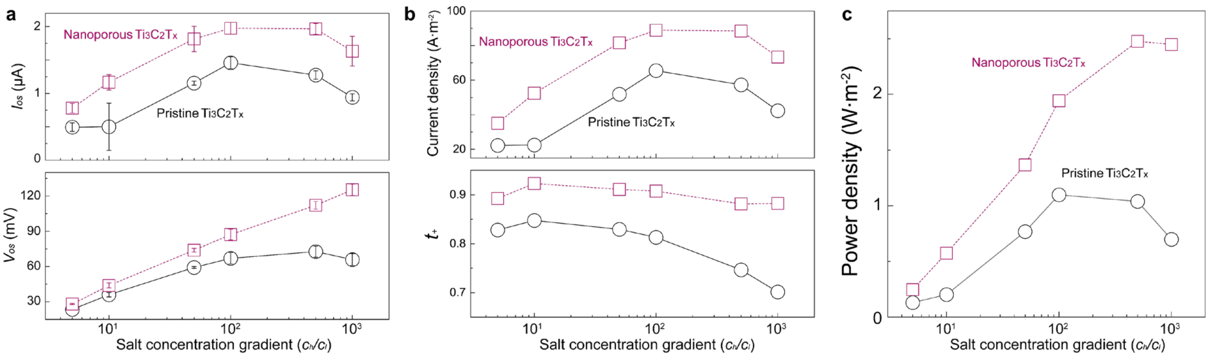

Figure S9 Concentration gradient-dependent osmotic performances across the thick nanoporous $Ti_3C_2T_x$ membrane with 9.26 μm thickness. The nanoporous membranes exhibit higher power densities of about 2.5 W/m^2 by up to 250 % compared to those of the pristine membranes with 9.28 μm thickness.

10. Chemical stability of nanoporous $Ti_3C_2T_x$ MXene membranes under aqueous operation

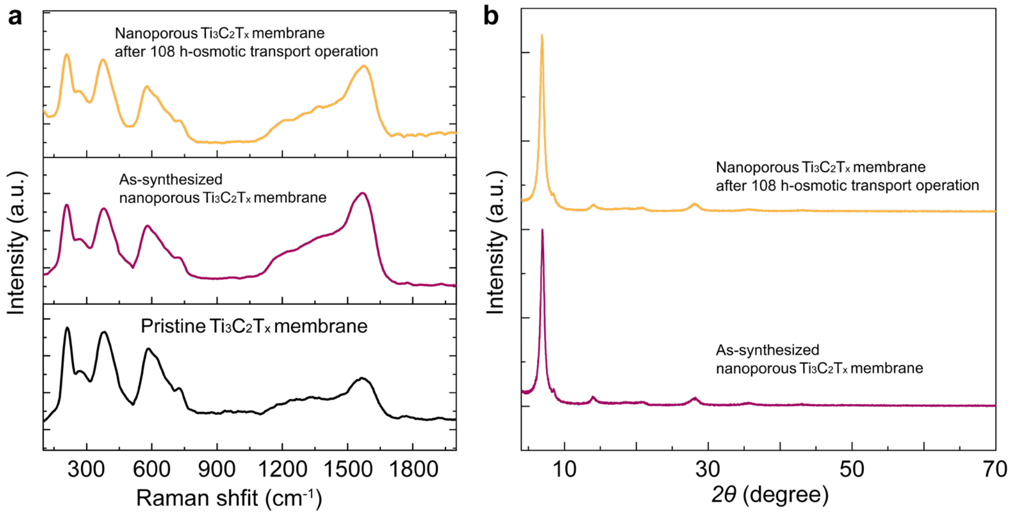

Figure S10 Aqueous chemical stability of the nanoporous $Ti_3C_2T_x$ MXene membranes over 100 hours **(a)** Raman spectra and **(b)** XRD patterns of the as-synthesized nanoporous MXene membranes and fully-dried nanoporous MXene membrane after 108 hours of the osmotic transport operation.

Supporting References

1. Hong, S.; Ming, F.; Shi, Y.; Li, R.; Kim, I. S.; Tang, C. Y.; Alshareef, H. N.; Wang, P., Two-Dimensional $Ti_3C_2T_x$ MXene Membranes as Nanofluidic Osmotic Power Generators. *ACS Nano* **2019,** *13* (8), 8917-8925.
2. Raidongia, K.; Huang, J., Nanofluidic Ion Transport Through Reconstructed Layered Materials. *Journal of the American Chemical Society* **2012,** *134* (40), 16528-16531.
3. Smeets, R. M. M.; Keyser, U. F.; Krapf, D.; Wu, M.-Y.; Dekker, N. H.; Dekker, C., Salt Dependence of Ion Transport and DNA Translocation through Solid-State Nanopores. *Nano Letters* **2006,** *6* (1), 89-95.
4. Wanunu, M.; Dadosh, T.; Ray, V.; Jin, J.; McReynolds, L.; Drndić, M., Rapid Electronic Detection of Probe-Specific MicroRNAs Using Thin Nanopore Sensors. *Nature Nanotechnology* **2010,** *5* (11), 807-814.
